# Increasing self- and desired psychiatric diagnoses among emerging adults: Mixed-methods insights from clinical psychologists

**DOI:** 10.1016/j.ijchp.2025.100661

**Published:** 2025-12-31

**Authors:** Matthias Neumann, Verena Steiner-Hofbauer, Martin Aigner, Anna Höflich, Anita Holzinger, Gloria Mittmann

**Affiliations:** a Research Centre Transitional Psychiatry, Karl Landsteiner University of Health Sciences, Dr. Karl-Dorrek-Straße 30, 3500 Krems, Austria; b Doctoral Programme Meduni Vienna, Medical University of Vienna, Spitalgasse 23, 1090 Vienna, Austria; c Teaching Center, Research Unit for Curriculum Development, Medical University of Vienna, Spitalgasse 23, 1090 Vienna, Austria; d Division of Psychiatry and Psychotherapeutic Medicine, University Hospital Tulln, Alter Ziegelweg 10, 3430 Tulln, Austria

**Keywords:** Self-diagnosis, Desired diagnosis, Social media, Assessment, Diagnostics, Malingering, ADHD, Autism

## Abstract

Anecdotal observations suggest that self-diagnoses and desired psychiatric diagnoses may be increasing among emerging adults, yet systematic evidence from clinical practice is scarce. This mixed-methods study surveyed 93 Austrian clinical psychologists (CPs) regarding their experiences with these phenomena in the context of conducting psychological assessments. CPs rated the frequency of both self-diagnoses and desired diagnoses as significantly higher than the neutral scale midpoint (“no change”), with large effect sizes (both *p* < .001). ADHD and ASD were most frequently identified as self-diagnosed or desired. Patients presenting with such expectations were commonly described as female, highly educated, and strongly engaged in online activities. CPs, many of whom indicated that they actively inquire about patients’ motives when suspecting a desired diagnosis, explained such pursuits mainly in terms of relief from guilt, identity affirmation, and social recognition, while treatment access was cited less often. Qualitative analyses highlighted three recurring themes: (1) the impact of self- and desired diagnoses on the course of the assessment itself, including diagnosis-driven responding and limited openness to collaborative exploration; (2) strong reactions to diagnostic discrepancies, such as emotional distress, rejection of outcomes, criticism of clinicians, or “diagnosis shopping”; and (3) increased demands on clinical practice, particularly extended assessment time and the challenges of feedback sessions where unexpected outcomes must be communicated with clarity and empathy. These dynamics are discussed in relation to online mental health cultures and the symbolic appeal of neurodivergence, underscoring how digital environments shape both the spread of self-diagnosis and the pursuit of professional confirmation.

## Introduction

In recent years, the phenomenon of self-diagnosis has received growing attention within mental health research, particularly in relation to younger populations with high levels of internet use (Armstrong et al., 2025; Rettew, 2024). Self-diagnosis is generally understood as the identification with, or belief in having, a psychological disorder without a formal evaluation by a mental health professional (Evans-Lacko et al., 2019; Narendorf et al., 2023). Studies suggest that self-diagnoses may have been increasing in recent years (Monteith et al., 2024). This could encourage help-seeking (Rutter et al., 2023), with recent findings indicating that around half of those experiencing mental health concerns eventually seek professional support (Sump et al., 2025). Digital platforms such as TikTok, YouTube, and Reddit but also the influence of TV series have increasingly been associated with self-diagnoses (Foster & Ellis, 2024; Karasavva et al., 2025; Mittmann et al., 2025; Zea Vera et al., 2022). Through content that often blends personal storytelling with diagnostic language, these platforms expose users to a wide range of psychological concepts, encouraging comparisons with one’s own experience. In some cases, individuals who recognize aspects of themselves in online mental health content go on to seek out a corresponding diagnosis from a professional (Milton et al., 2023). Having an official diagnosis can offer them not just validation, but also increased credibility and expert status within online peer groups (Giles, 2006).

From a developmental perspective, self-diagnosis appears to be closely linked to identity formation processes in adolescence and early adulthood as assigning oneself a diagnostic label can be experienced as a way of gaining insight, establishing coherence, or asserting agency in the face of difficulties (Harari et al., 2023; Zhang & Qin, 2023). However, strong identification with a particular label may promote self-essentialist thinking, reducing flexibility and reinforcing fixed self-concepts (Camp & Flores, 2024; Corrigan et al., 2009). Online mental health communities can facilitate a sense of connection and support (Chen & Xu, 2021; McCormack, 2010), especially for individuals who feel misunderstood or underserved by traditional care systems. In these contexts, self-diagnosis may serve not only as a form of meaning-making but also as an entry point into collective identity formation (Haltigan et al., 2023). Self-diagnostic online culture can therefore transform mental disorders into identity-forming experiences that foster a sense of belonging (Corzine & Roy, 2024), as evidenced by the frequent inclusion of psychiatric labels in the social media biographies of adolescents (Wiesböck, 2025). In online communities, self-diagnosing can become part of adopting a "sick role" as a way of making sense of distress that is often supported by empathy, encouragement, and a shared sense of belonging among peers (Haltigan et al., 2023). For example, a study on Tumblr showed that online-disclosure of illness on social media can generate social capital by eliciting support, attention, and recognition (Griffith & Stein, 2021). At the same time, not having a formal diagnosis can leave individuals feeling exposed to doubt or scepticism (Underhill & Foulkes, 2024), leading some to seek professional confirmation not only for clarity, but also as a way to strengthen their legitimacy and increase their standing within these online communities.

Considering these dynamics, it is highly plausible that some individuals seeking professional assessment do so with the hope of receiving a diagnosis that affirms an identity already shaped through self-diagnosis (Milton et al., 2023). When a particular label has become central to how a person understands themselves, alternative outcomes may be experienced as dissonant or invalidating. As public attitudes towards specific mental health diagnoses evolve, it is relevant to examine which conditions are most often viewed as socially acceptable or even desirable. Yet, it is important to note that the wish for receiving a particular diagnosis can also exist independently of prior self-identification. In this study, we refer to the diagnoses that individuals actively hope to obtain as “desired diagnoses.” Though the term desired diagnosis has not yet been formally defined in the literature, it can be understood as spanning a wide continuum of motivations for pursuing a diagnostic label. For some individuals, the wish for a particular diagnosis reflects a search for better self-understanding or coherence, consistent with processes of illness identity formation (Charmaz, 1995). For others, it resembles reassurance-seeking in the context of health anxiety, where professional confirmation is expected to reduce uncertainty or distress (Cougle et al., 2012; Parrish & Radomsky, 2010). In still other cases, the wish for a diagnosis may be linked to external or practical benefits, ranging from unintentional secondary gain to more deliberate symptom presentation for external incentives, as seen in malingering (Alozai & McPherson, 2025). Importantly, the term desired diagnosis is used here in a descriptive and value-neutral way, as it neither implies deliberate deception nor pathology, but simply denotes situations in which a particular diagnostic label as an outcome is preferred or hoped for by the patient, regardless of the underlying motive.

In the Austrian health care system, several professional groups are authorized to make mental health diagnoses. However, Clinical Psychologists (CPs) occupy a distinctive position in this landscape, as their assessments are typically designed to be especially comprehensive and methodologically structured, particularly when questions of differential diagnosis arise. To qualify for this role, individuals must first complete a university degree in psychology comprising 300 ECTS credits. This five-year program leads to a master’s degree and is followed by roughly two years of postgraduate clinical training. This training includes both theoretical instruction and closely supervised practical work. Once all requirements have been fulfilled, candidates can register with the Ministry of Health as CPs, which grants them the license to practice (Berufsverband Österreichischer PsychologInnen, 2025). To become eligible to work as a *Wahlpsychologe* or *Vertragspsychologe*, meaning a CP whose diagnostic services are fully (*Vertragspsychologe*) or partially (*Wahlpsychologe*) reimbursed by public health insurance, further qualifications are necessary. After registration, CPs must gain at least two years of professional experience and must also provide evidence of having independently conducted 100 diagnostic cases (Österreichische Gesundheitskasse, 2025). Notably, research has shown that CPs inquire about potential external incentives a lot more frequently than physicians (Aita et al., 2020), which further underscores their suitability as primary sources of information when studying desired diagnoses.

Some practitioners have noted anecdotally that an increasing number of clients arrive with the hope of receiving a specific diagnosis, often shaped by prior self-identification or online information. However, little is known about how such expectations are experienced in day-to-day clinical work. This study surveyed licensed CPs across Austria using both quantitative and qualitative methods. It explores how self- and desired diagnoses are encountered in practice and how these expectations may shape the assessment process. The main research questions (RQs) of the current study are as follows:

*RQ1: Do CPs in Austria perceive an increase in self-diagnosed and desired diagnoses in their diagnostic work?*

Based on previous literature and anecdotal observations by clinicians, we formulated two directional hypotheses relating to RQ1:

*H1: CPs report encountering self-diagnosed cases more frequently than in the past.*

*H2: CPs report that clients increasingly present with the goal of receiving a specific diagnosis.*

*RQ2: Which psychiatric diagnoses are most frequently desired or self-identified by patients, according to CPs?*

*RQ3: What motives do CPs attribute to patients who desire a specific diagnosis, and what motives do they perceive in patients who seek a professional diagnosis after already self-identifying with a condition?*

*RQ4: How do prior self-diagnoses and desired diagnoses shape the clinical diagnostic process?*

## Materials and methods

### Study design

A cross-sectional, internet-based survey was conducted from 15 June to 05 July 2025 using *SoSci Survey* (Leiner, 2024) and took approximately 15 min to complete. It was distributed via email to all CPs listed with the Austrian Federal Ministry of Social Affairs, Health, Care and Consumer Protection who had provided a valid email address (∼5500 out of >11,800 registered as of June 2025). Invitees were informed that participation was restricted to CPs conducting, on average, at least one assessment per week. About 10 % of the email addresses returned delivery failure notifications, typically due to inactive domains. Informed consent was obtained electronically, and participation was entirely voluntary and unpaid.

### Measures

Data were collected using an online survey containing both closed- and open-ended items. To assess perceived changes over time, CPs were asked whether they had noticed an increase or decrease in the frequency of patients presenting with either a self-diagnosed condition or a specific diagnosis they hoped to receive. Responses were recorded on a 5-point Likert scale (1 = “much less than before,” 2 = “less than before,” 3 = “same frequency as before,” 4 = “more often than before,” 5 = “much more often than before”), with an additional option of “I have never encountered this” for both items. CPs were also invited to describe, in an open-ended format, typical characteristics of patients most frequently presenting with self-diagnoses or desired diagnoses.

To identify the psychiatric conditions most commonly associated with these phenomena, respondents were presented with a checklist of common psychiatric diagnoses and asked to select all that applied for both self-diagnoses and desired diagnoses. An open-text “Other” field allowed respondents to list diagnoses not included in the predefined options.

Perceived patient motives were explored in two ways. First, CPs were asked about reasons they believed patients might wish to obtain a particular diagnosis. Second, they were asked about motives for seeking professional assessment after having already labelled themselves with a condition. For both sets of questions, CPs could choose from predefined lists (e.g., access to medical treatment, access to psychotherapy, relief from guilt or responsibility, or inclusion in a specific identity group) and were able to provide additional motives in a free-text field. CPs also indicated, on a 5-point Likert scale, how frequently they explicitly address patients’ motives when they suspect that a specific diagnosis is being sought, ranging from “never” to “always.”

Finally, the survey included four open-ended questions to capture qualitative insights into CPs’ experiences with these phenomena during diagnostic assessments. Participants were asked to describe notable patterns or situations they had encountered, challenges posed by patient-held diagnostic expectations, their approach when a patient’s self-diagnosis or desired diagnosis did not match their own clinical judgment, and memorable cases in which such a mismatch occurred. For the first of these questions, three predefined multiple-choice options were provided: (1) patients explicitly stating that they have already self-diagnosed themselves with a specific condition (2) patients explicitly stating that they wish to receive a very specific diagnostic label, and (3) patients bringing self-completed screening questionnaires from the internet. Many respondents elaborated in the “Other” category, and these additional examples were included in the qualitative analysis.

### Data analysis

#### Quantitative

Quantitative analyses were performed using R Statistical Software Version 4.4.1 [30]. Descriptive statistics were applied to participants’ demographic characteristics (age, gender, years of professional experience, and work setting), as well as to response distributions for the checklist items on self-diagnosed and desired diagnoses, and for the predefined lists of patient motives.

For the two hypotheses of RQ1, one-sample *t*-tests were conducted to test whether CPs perceived an increase in the frequency of self-diagnoses and desired diagnoses. Responses were recorded on a 5-point Likert scale (1 = “much less than before,” 2 = “less than before,” 3 = “same frequency as before,” 4 = “more often than before,” 5 = “much more often than before”), with an additional option of “I have never encountered this.” The latter was recoded to the scale midpoint (3 = “no change”), reflecting the assumption of no perceived change, so that all responses could be included in inferential analyses. Tests were conducted one-sided against the neutral scale midpoint (3 = “no change”), as the directional hypotheses specifically predicted increases in both self-diagnoses and desired diagnoses.

In addition to the planned one-sample *t*-tests, we conducted robustness checks to address the treatment of the response option “I have never encountered this” and the ordinal nature of the data.” First, we repeated the analyses after excluding respondents who selected “I have never encountered this” rather than recoding this option to the neutral midpoint. Second, we ran non-parametric Wilcoxon signed-rank tests treating the Likert ratings as ordinal rather than interval-scaled. Finally, we conducted exploratory chi-square goodness-of-fit tests to examine whether the observed response distributions differed from a uniform pattern. Effect sizes are reported as Cohen’s *d*.

#### Qualitative

Inductive content analysis (Kiger & Varpio, 2020) was conducted without a predefined framework or coding scheme. The analysis focused on participants’ responses to several open-ended questions addressing their clinical experiences with self-diagnoses and desired diagnoses. Responses were thematically examined to identify recurring patterns and explanatory models as well as professional reflections. Areas of focus included perceived reasons for their perceived increase or decrease in self- and requested diagnoses over time, descriptions of individuals presenting with self-diagnoses (e.g., demographic or psychosocial patterns), and observed challenges associated with patients’ diagnostic expectations. Participants were also asked to report phenomena encountered in clinical practice related to self- or desired diagnoses, as well as frequently observed discrepancies between patients’ self- or desired diagnoses and clinically determined diagnosis. Additionally, the analysis examined clinicians' handling of situations in which patient-generated diagnoses diverged from professional assessment, the perceived influence of such prior expectations on the diagnostic process, and reflections on the benefits and drawbacks of addressing self- or requested diagnoses within the diagnostic setting. Especially noteworthy or illustrative examples, including memorable cases and common patient responses, were also examined using thematic analysis.

Thematic analysis began with a detailed review of the data to ensure familiarity. Two researchers (MN and GM) carried out the coding. Both are trained psychologists with experience in qualitative research. MN is a clinical psychologist working in diagnostic settings and a pre-doctoral researcher; although prior contact with individual respondents cannot be fully excluded, anonymity prevented any identification. GM is a post-doctoral academic psychologist with no clinical link to participants. The coders independently coded the first 40 % of responses, generating initial codes that captured key topics. These were then compared, discussed, and refined into broader themes through an iterative process involving regular meetings and revisions. After reaching satisfactory inter-coder agreement (Cohen’s Kappa > 0.70), the remaining data were coded using the finalized framework, with regular checks to ensure consistency. Data saturation was reached, as no new themes emerged in the final portion of the dataset. Responses addressing multiple topics were assigned to all relevant themes.

## Results

### Sample

The study sample consisted of 93 CPs. The mean age was 48.4 years (*SD*=10.6), and 89.3 % of participants identified as female. This gender distribution seems broadly in line with the Austrian workforce. While no current nationwide figures exist for licensed clinical psychologists, recent data show that about 85–87 % of clinical psychology trainees are women (Sagerschnig et al., 2025). Participants had an average of 16.3 (*SD*=8.8) years of professional experience. Among the participants, 22.58 % were employed under a full public health insurance contract offering full reimbursement, 15.1 % practiced as elective psychologists with partial reimbursement, and 62.4 % worked exclusively in private practice. In addition to their roles in the outpatient sector, 24.7 % of participants were also employed in hospitals, and 22.6 % in other professional institutions.

### RQ1: Do CPs in Austria perceive an increase in self-diagnoses and desired diagnoses in their diagnostic work?

Self-diagnosis appears to be an increasingly frequent phenomenon, according to the surveyed CPs. Specifically, 21 (22.6 %) indicated this occurs “much more often than before” and 47 (50.5 %) selected “more often than before.” A further 21 (22.6 %) perceived no change in frequency, while four participants (4.3 %) stated they had never encountered this phenomenon. Full response distributions are available in Supplementary Table S2. The result indicated a statistically significant increase, *t*(92) = 13.08, *p* < .001, with a mean of 3.96 (*SD* = 0.71), 95 % CI [3.84, ∞), and a large effect size (*d* = 1.36), supporting the directional hypothesis.

**Fig. 1:**
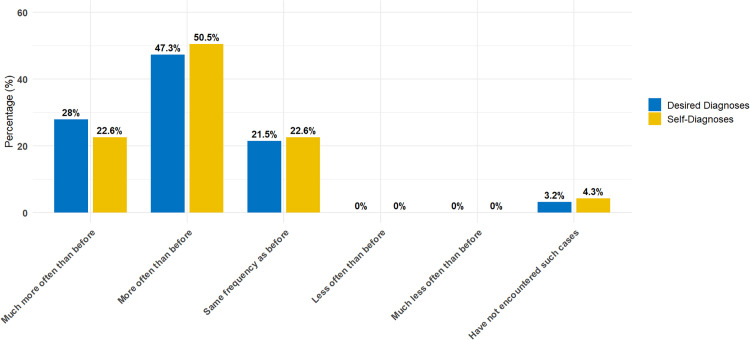
Clinical Psychologists’ Perceptions of How Often Self- and Desired Diagnoses Occur Today Compared to the Past.

Reports of patients increasingly seeking to attain a desired diagnostic label were frequent among the surveyed CPs (see Fig. 1). In total, 26 (28.0 %) indicated this happens "much more often than before" and 44 (47.3 %) indicated this happens "more often than before". In comparison, 20 (21.5 %) felt the frequency has remained the same. No participant indicated a decrease in the occurrence of desired diagnoses. Three participants (3.2 %) stated they had not encountered such cases at all. A one-sided one-sample *t*-test against the neutral midpoint (3 = “no change”) indicated a significant increase in the perceived frequency of desired diagnoses, *t*(92) = 13.65, *p* < .001, *M* = 4.03, *SD* = 0.73, 95 % CI [3.91, ∞), *d* = 1.42. To assess the directional hypothesis that desired diagnoses have become more common, a one-sided one-sample *t*-test was performed. The findings revealed a statistically significant rise, *t*(92) = 13.65, *p* < .001, *M* = 4.03 (*SD* = 0.73), 95 % CI [3.91, ∞), and a large effect size (*d* = 1.42), providing evidence in support of the directional hypothesis. All robustness analyses confirmed the original pattern of results. Wilcoxon signed-rank tests, analyses excluding the “never encountered” responses, and chi-square goodness-of-fit tests all remained statistically significant in the same direction (see Supplementary Table S3).

Qualitative analysis of the open-ended format showed that typical characteristics CPs observe in patients who either seek a specific diagnosis or present with a self-diagnosis overlapped substantially. Notable are the three most frequently mentioned characteristics: Of the 93 participants, 18 and 22 (for self-diagnosis and desired diagnosis respectively) mentioned being “female”, 13 and 22 mentioned having a "higher education", and 8 and 17 mentioned engaging in "high online activity" to be characteristics of their patients. A complete overview of all reported characteristics is provided in *Supplementary Table 1*.

### RQ2: Which psychiatric diagnoses are most frequently desired or self-identified by patients, according to CPs?

**Fig. 2:**
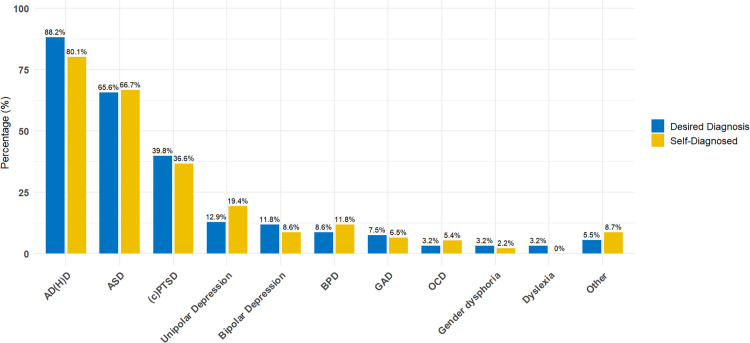
Diagnoses patients commonly desire or have already self-diagnosed according to Clinical Psychologists. Note. AD(H)D = Attention Deficit (Hyperactivity) Disorder, ASD = Autism Spectrum Disorder, (c)PTSD = (Complex) Post-Traumatic Stress Disorder, BPD = Borderline Personality Disorder, GAD = Generalized Anxiety Disorder, OCD = Obsessive-Compulsive Disorder.

CPs’ responses revealed a high degree of overlap between desired and self-diagnosed conditions. Attention deficit hyperactivity disorder (ADHD) and autism spectrum disorder (ASD) emerged as the most commonly cited diagnoses by far, with 83 (88.2 %) CPs reporting that patients often wish to receive an ADHD diagnosis, and 62 (66.7 %) noting this for ASD. Self-diagnosis patterns followed a similar trend, with 74 (79.6 %) CPs reporting that patients had frequently already identified themselves as having ADHD, and 63 (67.7 %) for ASD. Mentions of additional diagnoses were comparatively rare and are shown in Fig. 2.

### RQ3: What motives do CPs attribute to patients who desire a specific diagnosis, and what motives do they perceive in patients who seek a professional diagnosis after already self-identifying with a condition?

**Fig. 3:**
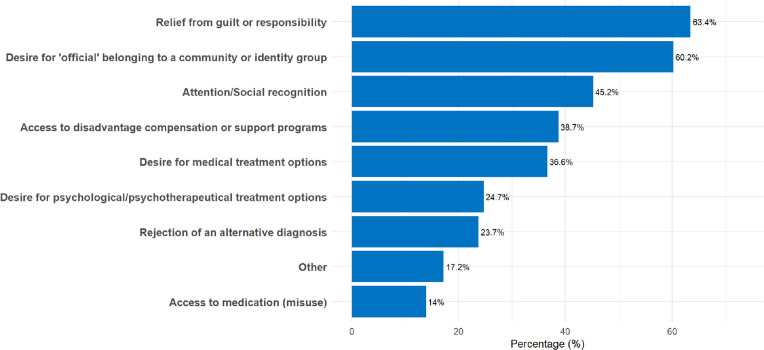
Clinical Psychologists’ observations of motives behind receiving the specific desired diagnosis.

As for the motives attributed to patients seeking a specific diagnosis, CPs most often highlighted relief from guilt or responsibility, the desire for formal inclusion in a particular identity group and attention/social recognition. Other motives, like gaining access to treatment possibilities, were reported less often (see Fig. 3).

**Fig. 4:**
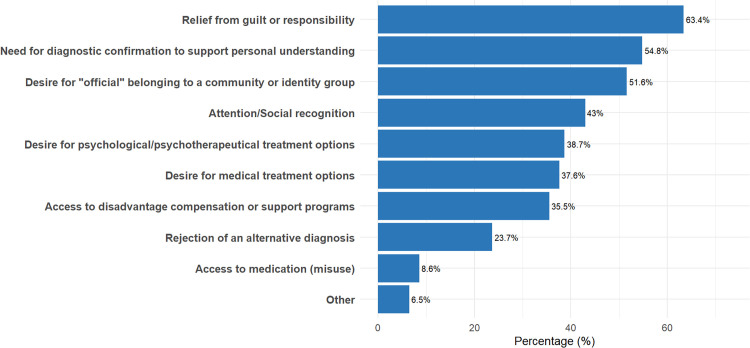
Clinical Psychologists’ observations of patient motives for pursuing professional diagnosis post self-diagnosis.

As with patients desiring a specific diagnosis, CPs reported that those seeking confirmation after self-identifying most often did so for relief from guilt or responsibility and to validate their self-understanding. Identity affiliation and social recognition were again common, with treatment-related motives appearing less frequently (see Fig. 4).

To clarify how CPs come to understand patients’ diagnostic motives, they were asked whether they raise the topic when they suspect a specific diagnosis is being sought. Quantitative data suggests that this appears to be a regular part of diagnostic conversations: 28 (30.1 %) said they always bring it up in such cases, and 24 (25.8 %) indicated they often do, even when their impression is minimal. Another 28 (30.1 %) said they do so occasionally. In contrast, 6 (6.5 %) reported raising the issue only when they had a particularly strong suspicion of a desired diagnosis, and 2 (2.2 %) stated they never do. Five (5.4 %) had not encountered a specific situation in which they suspected a desired diagnosis in their practice.

### RQ4: How do prior self-diagnoses and desired diagnoses shape the clinical diagnostic process?

CPs were asked whether they had encountered any of the following three scenarios in their clinical work. Nearly half (46.2 %, *n* = 43) reported that patients had brought self-completed diagnostic questionnaires from the internet to the assessment. A similarly high proportion (45.2 %, *n* = 42) indicated that patients explicitly stated they had already self-diagnosed themselves with a specific condition. Slightly fewer, but still a substantial proportion (41.9 %, *n* = 39), reported cases in which patients explicitly declared that they wished to receive a particular diagnosis. Through inductive content analysis we identified three broad themes (see Table 1), which are the (1) the impact of prior knowledge and expectations on the course of assessment itself, (2) patients’ reactions to diagnostic discrepancies, and (3) additional demands placed on clinical practice. A small number of codings (*n* = 3) could not be meaningfully integrated into these categories and were therefore not reported further.

**Table 1:** Themes, subthemes and coding frequencies (*N* = 359 codings).

Theme/Subtheme		Definition	*n* (Codings)
**Theme 1: Impact on the Course of Assessment**			
1.1 Informed Prior (Half-)knowledge	Patient reliance on partial or distorted knowledge from online sources, social media, or peers.		42
1.2 Response Patterns in Diagnostic Procedures	Biased or diagnosis-driven responding in interviews and questionnaires.		19
1.3 Resistance to Collaborative Exploration	Predetermined stance limiting openness to alternative explanations during exploration.		8
**Theme 2: Reactions to Diagnostic Discrepancies**			
2.1 Rejection of Diagnostic Outcome	Dismissal of clinical conclusions when anticipated diagnosis not confirmed.		57
2.2 Affective Responses	Emotional reactions such as disappointment or anger.		42
2.3 Critiques of Clinicians and the Diagnostic Procedure	Criticism directed at clinicians or questioning of assessment validity.		33
2.4 Coercive Strategies	Attempts to pressure clinicians through threats or manipulation.		24
2.5 Diagnosis Shopping	Consulting multiple providers until the desired diagnosis is obtained.		6
**Theme 3: Practice Demands**			
3.1 Balancing Empathy and Professional Authority	Need to combine empathic communication with clear professional boundaries.		90
3.2 Increased Resource Demands	More time and effort is required in such assessments.		38

### Theme 1: impact on the course of assessment

CPs reported that in cases where patients arrived with a self-diagnosis or a desired diagnosis, the diagnostic process was noticeably shaped by these prior expectations. Instead of entering the assessment primarily to seek professional advice, many patients came with a clear idea of the diagnosis they wanted to receive. CPs described this shift as altering the balance of the clinician–patient interaction. Three aspects were particularly prominent: the type of knowledge patients brought with them, the way they completed diagnostic procedures, and the general attitude with which they approached the assessment.

### Subtheme 1.1: informed prior (Half-) knowledge

Patients often came with extensive background information gathered from social media, online self-tests, or opinions from friends or family members. While some of this preparation reflected genuine interest and engagement, CPs highlighted that it frequently included psychological half-knowledge with selective or distorted understandings of psychiatric criteria. This sometimes led patients to over-pathologize everyday experiences or interpret subclinical difficulties as clear-cut signs of a disorder.

*“Most patients do not have a realistic understanding of what a ‘healthy psyche' looks like. When asked about a short attention span, they answer ‘yes,’ but when probed further—for example, how long they can concentrate during a lecture—they indicate one to two hours.”*

*“I heard my symptoms being described on Instagram and followed the link. The strategies there helped me, so I’m sure I have it too.”*

*“Even in the initial email request, patients sometimes write things like, ‘I have been dealing with ADHD and autism for years and completed all the screening tools, now I just want confirmation,’ or ‘I have completed all the screenings and gathered statements from family and friends, which I can bring along.’”*

### Subtheme 1.2: response patterns in diagnostic procedures

A common challenge described by CPs was that diagnostic procedures, particularly self-report questionnaires and interviews, were filled out in ways that reflected the diagnosis patients already had in mind. This diagnosis-driven way of responding complicated the task of distinguishing between actual symptomatology and malingering. In many cases, third-party assessments or school reports did not align with the patient’s self-report.

*“The hardest part is how to make sense of self-reports when a person clearly wants a diagnosis and, due to their education, has access to the criteria beforehand. Often, third-party histories are needed, where parents then report that the patient was completely calm and inconspicuous as a child and school records confirm this, while the retrospective self-report paints a very different picture.”*

*“Patients often inform themselves extensively about symptoms and disorders beforehand, pathologize more experiences, and answer both interviews and questionnaires accordingly.”*

### Subtheme 1.3: resistance to collaborative exploration

Finally, CPs noted that patients with a self-diagnosis or desired diagnosis sometimes approached the interaction with a predetermined stance. This attitude limited the openness to critical evaluation on the side of patients, leaving little room for collaborative consideration of alternative explanatory models. As one CP noted, patients sometimes entered the assessment saying, *“I already know I have it; I just need it officially confirmed.”* Another CP reported encountering a “*biased and hostile attitude*” toward the evaluation process.

### Theme 2: reactions to diagnostic discrepancies

When providing diagnostic feedback to patients who had entered assessments with a prior self-diagnosis or desired diagnosis, clinicians frequently reported difficulties once patients learned that the evaluation results did not match their expectations. Five interrelated themes characterized the spectrum of patient responses to diagnostic mismatches: (1) non-acceptance of the clinician’s diagnosis, (2) emotional reactions, (3) criticism of the clinician or the diagnostic process, (4) overt or covert pressure to adjust the diagnosis, and (5) “diagnosis shopping.”

### Subtheme 2.1: rejection of diagnostic outcome

In many cases, patients strictly resisted the diagnostic outcome when it diverged from their anticipated self- or desired diagnosis. CPs described situations where patients demanded that alternative diagnoses be removed from reports or explicitly asked for the desired diagnosis to be given despite not meeting the criteria:

*"The peak moment was when a female patient said after the feedback session: 'I now understand that I don't meet the criteria for ADHD, but could you please give me the diagnosis anyway?' When I asked what would change for her by receiving a diagnosis which she does not fulfil the criteria for, she said that she would also like to be 'diverse in some way or another.'"*

Some patients also challenged clinical judgment by referencing artificial intelligence, as one clinician reported: *"Instead of ADHD, I gave a Bipolar Disorder diagnosis. This was vehemently denied by the patient because the AI says something different."* Another CP experienced a *"Rejection of the findings and subsequent refusal to exit the meeting room (a three-hour closing discussion and eventual need for someone to pick them up)”.*

### Subtheme 2.2: affective responses

Strong emotional responses were frequently described when patients were confronted with a mismatch between their anticipated diagnosis and the CP’s diagnosis. While some patients experienced relief when their self-diagnosis was not confirmed, the majority of emotional reactions involved distress when expectations were not met. These reactions ranged from deep disappointment and sadness to feelings of being misunderstood or rejected. Some patients expressed distress at having to reconsider their self-concept or identity in the absence of the expected diagnosis, while others displayed frustration or resignation.

*"On one occasion, a patient began crying heavily and then admitted that they had already been to two other clinical psychologists for assessment and was now being diagnosed with a personality disorder for the third time instead of the desired ADHD. However, the patient was able to be calmed down and eventually came to terms with the diagnosis."*

### Subtheme 2.3: critiques of clinicians and the diagnostic procedure

CPs also reported being confronted with criticism after presenting their diagnostic conclusions that did not align with patients’ expectations. This criticism was directed both at them personally and at the assessment process more generally. Examples included negative online reviews and challenges to the validity of the used diagnostic methods themselves. In extreme cases, patients exhibited severe behavioral dysregulation, as described by another CP: *'Aggressive outburst, loss of emotional control, verbal abuse, completely inappropriate behavior, stormed out of the practice and came back 3 times to continue dominating.'* This same patient later declared it a form of *'punishment - all psychologists are severely disturbed themselves - I'm never coming back here.'*"

### Subtheme 2.4: coercive strategies

CPs described being subjected to direct or indirect pressure to issue a particular diagnosis. Such pressure took various forms, including threats of legal action, refusal to pay fees, or demands to revise written reports. One clinician described how *"The patient wanted an ADHD diagnosis, but it was a personality disorder. The patient reacted with aggression and threats (lawsuit), etc."* Financial coercion was also reported, with patients issuing a *"threat not to pay"* when desired diagnoses were not provided. In some cases, the pressure extended far beyond the clinical encounter, as one CP experienced: *"A patient who rewrote my report after 5 years and said I had to revise it accordingly within 2 weeks, otherwise she would sue me."* In extreme cases, clinicians even reported vandalization or harassment, with one CP reporting that *"'a colleague had ‘Nazi bitch’ spray-painted on her office door with a spray can"* after declining to issue the diagnosis requested by the patient. Some patients employed emotional manipulation, with one stating *"that the potential diagnosis had been the only reason so far for not having committed suicide."*

### Subtheme 2.5: "diagnosis shopping"

Another pattern involved patients seeking out multiple diagnostic evaluations by different CPs until they obtained the label they were hoping for. One CP reported frequently hearing: *"I know that I have this. If I don't get the diagnosis, I'll have to find someone more competent."* Clinicians observed that some patients seemed to learn from each assessment experience, refining their self-reports to better align with the desired diagnosis in subsequent encounters.

*"Sometimes diagnostic shopping can be observed. Experience is gathered regarding a specific diagnosis, and if the diagnosis is not given, the patients go to the next psychologist until they receive it. The better they know what they need to answer, the higher the probability that they get a diagnosis."*

*“They just go to other assessments until they get what they want (one even sent me the report saying 'See! I do have it, you just couldn't recognize it!' (interestingly, the self-report about childhood in the WURS questionnaire was completely different than with me… So a 'learning effect' can be assumed when people don't get their desired diagnosis and simply move on until they get it (they can see from the summary or feedback session why they didn't get what they wanted)"*

### Theme 3: modified practice demands

Clinicians emphasized that when patients entered the assessment with a self- or desired diagnosis, this placed particular demands on how the evaluation was communicated and managed. Two recurring aspects were highlighted: the need for very sensitive yet also factual and transparent communication, and the increase in time and effort required to reach an accurate diagnosis.

### Theme 3.1: balancing empathy and professional authority

An important strategy described by CPs was to engage in thorough and empathic communication. Clinicians reported that it was often necessary to carefully explain what often-desired psychiatric diagnoses such as ADHD or ASD actually mean, and to clarify step by step why the anticipated label was not confirmed in a specific case. This included discussing the reasoning behind alternative diagnoses as well as providing psychoeducation concerning the assessment process itself. One CP particularly emphasized the importance of transparency: *"Transparently discussing what diagnostics means and does takes pressure off expectations and establishes that my work is based on their answers and my expertise, and by no means on their expectations."* Another described their approach: *"I explain that I have tests available to me that are based on science, and I have guidelines to use them. I have years of experience and from this, I try to approach diagnosis as objectively as possible and create the most comprehensible diagnosis from history and testing."* Several CPs noted that framing the discussion in terms of professional expertise and diagnostic standards helped reduce the expectation that the outcome could be “negotiated”. However, many described that addressing the issue with empathy was equally important, as for some patients the desired diagnosis had become tied to questions of identity and belonging. As one CP explained: *"I still give my diagnosis, but you really have to be empathetic during the feedback session… for many this is an identity search, and not getting the desired identity (where nowadays people are told they are exactly what they identify as) is very hurtful."* When approached with care, positive outcomes were possible: *"With empathetic and well-founded explanation and education about what diagnoses (ADHD/ASD) mean, how I arrive at the alternative diagnosis, why the desired diagnosis is not present, and what treatment options exist for the alternative diagnosis, this is usually very well understood and accepted."*

### Theme 3.2: increased resource demands

CPs reported that working with patients who arrived with self-diagnoses or desired diagnoses required substantially more time throughout the entire diagnostic process. The additional effort began during the diagnostic evaluation itself, as clinicians needed to probe more thoroughly during history-taking when patients had preconceived diagnostic expectations. Information was often not readily disclosed when desired diagnoses were present, requiring more detailed questioning and follow-up. Additional external assessments, school reports, and collateral information frequently needed to be obtained to ensure diagnostic accuracy in such cases. When patients openly communicated that they had a desired diagnosis, clinicians also had to invest time in exploring the underlying motivations and expectations. Additionally, diagnostic feedback sessions often turned into lengthy discussions, as clinicians had to address misunderstandings, counter resistance, and revisit patients' expectations. The scope of this challenge was considerable, as one CP points out: *"It takes double the time, actually multiple clinical-psychological sessions are necessary."* Some CPs noted that entire sessions were needed just to manage disagreements or to prepare patients for outcomes that did not align with their hopes, with one noting that in cases of previous self- or desired diagnosis *"Every feedback session turns into a discussion, that's when the victim role comes into play again."* Others described the emotional toll of anticipating conflict and having to enter such encounters mentally prepared: *"Before the session, gathering a lot of energy, being prepared for negativity".*

## Discussion

The present study aimed to examine current trends in self-diagnosis and desired diagnoses among emerging adults from the perspective of CPs. In recent years, there have been reports of an increase in self-diagnosis and related behaviors (Gilmore et al., 2022; Monteith et al., 2024; Rettew, 2024). However, to the best of our knowledge, our study is the first to examine whether a large sample of mental health diagnosticians perceives such an increase in their clinical practice. Consistent with anecdotal reports, our findings indicate a clear increase in both phenomena. The majority of CPs reported encountering emerging adult clients who had already self-identified with a psychiatric condition more frequently than in the past, and an equally strong trend emerged for patients explicitly seeking a particular diagnostic label. Statistical analyses confirmed that both self-diagnoses and desired diagnoses were perceived as occurring significantly more often than in previous years, with large effect sizes supporting these impressions. The results point to patient-generated diagnostic expectations as an increasingly salient issue in the assessment work carried out by CPs.

CPs most frequently described individuals in this group as female, highly educated, and engaging in intensive online media use. This profile aligns with the notion that greater exposure to digital mental health content may increase both the likelihood of self-identifying with psychiatric labels and the tendency to pursue desired diagnoses. Research has repeatedly highlighted that social media platforms such as TikTok (Foster & Ellis, 2024), Instagram (Bailie, 2024), and Tumblr (Griffith & Stein, 2021) have become central arenas for the circulation of self-diagnosis content and mental health narratives. Phenomena such as “TikTok’s sick-role subculture” (Harness & Getzen, 2022) may amplify the visibility of psychiatric labels and may encourage their social adoption or even social contagion. Furthermore, CPs’ frequent reference to female patients aligns with previous observations that social contagion of psychiatric conditions has historically been more prevalent among women (Allison et al., 2013; Hermansson-Webb, 2014). The ongoing fusion of self-diagnosis culture and social media trends may therefore contribute to social contagion, reinforcing the appeal of diagnostic identification and increasing the salience of both self- and desired diagnoses in clinical practice.

The present findings further point to a substantial overlap between the diagnoses that emerging adults most frequently self-apply and those they actively wish to receive. ADHD and ASD clearly dominate both categories, standing out as the conditions most often mentioned by CPs. This alignment suggests that self-diagnoses and desired diagnoses tend to revolve around a small number of disorders, with few other diagnoses mentioned frequently. Successful advocacy efforts have contributed to reducing stigma surrounding diagnoses such as ADHD and ASD, which in online spaces are increasingly discussed under the broader umbrella of “neurodivergence” (Avelar, 2024; Steiner-Hofbauer et al., 2025; The Society for ADHD, 2025). At the same time, this shift might have fostered a cultural reframing in which these conditions are portrayed as conferring special abilities or even a form of social distinction. For instance, ADHD is sometimes described as being associated with “superpowers” on activist-oriented websites (eg., ADHD-Naturally, 2025; Burch, 2023; Good Mind & Body, 2024), a term also spontaneously used by a participant in a recent qualitative study to describe his ADHD (Frick et al., 2025). In the case of ASD, media portrayals frequently overemphasize links to savantism (Mittmann et al., 2024). Such portrayals of neurodivergent identities may inadvertently enhance the symbolic appeal of these diagnoses, potentially making them attractive as identity markers and possibly contributing to their prominence among desired diagnoses in clinical practice. This might further be reinforced by the fact that unlike disorders typically characterized by episodic or remitting trajectories, ADHD and ASD are defined as enduring neurodevelopmental conditions, making them particularly well suited to function as stable identity markers.

In line with the growing identity-related framing of ADHD and ASD, the motives most frequently described by CPs emphasized social and self-concept benefits over clinical treatment needs. Emerging adults often sought diagnoses to relieve feelings of guilt or inadequacy, particularly the sense of struggling to manage everyday responsibilities. This aligns with the view that ADHD can lend itself as a compelling interpretive framework for perceived inefficiency or difficulties in meeting everyday demands by offering a socially recognizable explanation (Wiesböck, 2025). Historically, categories such as “hysteria”, later reframed as neuroses, provided a recognized framework within medicine for forms of suffering that otherwise seemed elusive (Stone et al., 2008). The seeking of a specific “desired diagnosis” might serve a related purpose, helping individuals themselves to frame vague feelings of inadequacy in terms that resonate socially. In both cases, the transformation of indeterminate distress into named categories facilitate a sense of coherence and legitimacy. Social media communities further reinforce this dynamic by circulating narratives that oscillate between justified recognition and problematic overidentification (Hasan et al., 2023; Oehler, 2024). In addition, many appeared to pursue diagnostic confirmation as a way to affirm belonging to a condition-specific social group and to gain attention or recognition from others. This dynamic aligns with observations from studies of online mental health communities, where formal diagnoses often carry considerable weight, even in spaces that position themselves as critical of professional psychiatry (Giles & Newbold, 2011). By contrast, traditional treatment-related motives such as accessing medication or psychotherapeutic support were much less frequently reported. This imbalance may reflect that, for some patients, the primary goal is not to address the underlying difficulties but to acquire a socially recognized explanation for them. The diagnostic label itself can thus serve as a form of psychological relief, offering legitimacy and an externalized reason for why daily functioning feels challenging. Furthermore, research on need for cognitive coherence (Brannon & Gawronski, 2018; Gawronski & Brannon, 2019) suggests that diagnostic labels could reduce ambiguity and provide an organizing framework for otherwise diffuse experiences (Sims et al., 2021). Likewise, self-verification theory likewise suggests that individuals seek external confirmation of self-beliefs, which may increase the desire for a diagnosis that aligns with an already internalized self-concept (Swann, 1983). This perspective may also help explain the strong overlap observed in our data between self-diagnoses and desired diagnoses, because once a diagnostic identity has been internally adopted, the pursuit of a formal diagnosis could operate as a self-verifying step that secures external validation for an identity that is already psychologically in place. In addition, individuals with high intolerance of uncertainty, a transdiagnostic factor that is well documented in anxiety and depressive disorders (Gentes & Ruscio, 2011; Morriss, 2025), may be especially inclined to seek diagnostic clarification as a way of reducing ambiguity and psychological tension. For such individuals, receiving a definitive label such as ADHD or ASD may function not only as an explanation but as a form of cognitive closure, since neurodevelopmental diagnoses imply stability and therefore reduce future uncertainty. This may help explain why individuals with high intolerance of uncertainty might potentially be more motivated to pursue an ADHD diagnosis rather than one associated with fluctuation or remission, such as an anxiety disorder. This, however, carries the clinical risk of shifting the focus away from anxiety disorders, which could become under-recognized if the diagnostic process becomes centered on confirming ADHD. This is particularly relevant given that, though some evidence suggests elevated uncertainty intolerance in ADHD (Gramszlo et al., 2018), the phenomenon is far more robustly established as a core feature of anxiety disorders and depression (Jensen et al., 2016; Morriss et al., 2024; Yao et al., 2022).

It is worth noting that CPs’ impressions of patient motives are grounded in direct clinical conversations rather than mere assumptions. Many respondents indicated that they raise the topic of a patient’s motives as soon as they have any suspicion that a desired diagnosis might be involved, a practice consistent with prior research showing that CPs often probe for potential incentives (Aita et al., 2020).

According to CPs, the motivational patterns among individuals who sought professional assessment after having already self-diagnosed were highly similar to those observed for desired diagnoses. Clinicians most often perceived motives related to relieving guilt or responsibility and to achieving social or identity-related validation. Once again, CPs reported that treatment-related goals were far less prominent, reinforcing the impression that many of these encounters are driven primarily by the search for external legitimation and identity stabilization rather than by a focus on symptom alleviation. In accordance with that, within Gen Z, mental diagnoses have even been discussed as functioning like status symbols (Ahuja & Fichadia, 2024). This emphasis on receiving the desired label aligns with observations from online mental health communities, where professionals are valued primarily as authoritative gatekeepers whose formal recognition confers status, while their therapeutic expertise is often doubted (Giles & Newbold, 2011).

Beyond these social and identity-related dynamics, the findings also carry direct implications for clinical practice, particularly in light of the limited diagnostic accuracy of ADHD self-report measures. A recent systematic review demonstrated that while these scales generally achieve very high negative predictive values, their positive predictive values remain low, in most cases falling below 20 % (Harrison & Edwards, 2023). On top of these general limitations of self-report questionnaires, their reliability becomes even more questionable at a time when patients actively pursue particular diagnoses. In these cases, external sources like third-party ratings or school reports (Waltereit et al., 2025) offer perspectives that are harder to manipulate and could therefore be given greater weight in validating or counterbalancing subjective reports.

The qualitative results highlight how self- and desired diagnoses can shape clinical assessments. CPs reported that many patients arrive with considerable “half-knowledge,” often derived from online sources, which sometimes leads to distorted understandings of psychiatric disorders. This aligns with the quantitative result that patients with diagnostic expectations frequently report high online activity, and it resonates with prior studies showing that social media mental health content – particularly regarding ADHD and ASD – is often misleading (Aragon-Guevara et al., 2025; Brown et al., 2024; Karasavva et al., 2025; Verma & Sinha, 2024; Yeung et al., 2022). Another recurring theme was patients moving on to other CPs when the desired diagnosis was not confirmed. We refer to this pattern as “diagnosis shopping,” in analogy to the well-documented phenomenon of “doctor shopping” (Ng et al., 2020; Sansone & Sansone, 2012), with the crucial difference that the sought-after outcome is not a specific treatment but receiving a desired diagnostic label. The intense emotional reactions to diagnostic discrepancies may also be read as manifestations of contested definitional authority – conflicts over who is entitled to decide whether someone “truly” has ADHD or ASD (Aragon-Guevara et al., 2025; Hens et al., 2019). If these categories are experienced by patients as central aspects of identity, it is understandable that the refusal of such a label evokes far stronger affect than the routine medical communication of a given disorder being absent. In accordance with that, CPs emphasized that feedback sessions require particular sensitivity. Many reported that empathetic communication is indispensable when explaining why a desired label was not given, especially since some patients connected their diagnostic expectations closely with questions of identity and belonging. While empathy is widely recognized as a central therapeutic factor in psychotherapy (Elliott et al., 2018; Nienhuis et al., 2018; Vitinius et al., 2018), our findings underline its crucial role during diagnostic feedback in clinical assessments. Based on these findings, it would be valuable for clinical psychology training to explicitly address how to manage patient-held diagnostic expectations. This could include ways of responding to patients who arrive with desired or self-diagnoses, how to explain diagnostic reasoning clearly and respectfully, and how to manage situations in which a patient insists on a particular label.

### Strengths and limitations

To our knowledge, this is the first study to explicitly survey a large sample of CPs regarding perceived trends in self-diagnosis and desired diagnoses among emerging adults. A notable strength lies in the study’s broad, nationwide sample, which included practitioners from diverse settings, allowing for a more comprehensive picture of how these phenomena are perceived across Austrian psychological practice. Focusing on CPs as key informants for diagnostic processes offers valuable insight into patient-driven expectations within the context of in-depth psychological assessments. The study’s joint consideration of self-diagnosis and desired diagnoses provides a unique vantage point for understanding these overlapping trends and their implications for both clinical work.

Despite these strengths, several limitations warrant consideration. First, the findings reflect the perspectives of Austrian CPs and may not fully generalize to other countries given the cultural differences in stigma and public discourse around mental health (Ahad et al., 2023; Yusuf et al., 2024). Second, our results are limited to the viewpoint of a single professional group involved in mental health diagnostics. While CPs play a central role in formal assessments, other professionals such as psychiatrists, psychotherapists, and general practitioners may encounter different dynamics and trends in patient behavior. Third, the study solely relied on self-reported perceptions of CPs, which are shaped by clinical experience and interactions but do not represent direct patient data. It should be noted that CPs’ perceptions may also be influenced by recent media visibility of ADHD and ASD, which could amplify the sense of an increasing trend. Fourth, the sociodemographic information reported in this study is based on clinicians’ subjective impressions rather than standardized patient-level data. Future research should therefore include structured sociodemographic and psychological measures in order to more precisely characterize individuals who present with self- or desired diagnoses and to identify potential predictors of these behaviors. Fifth, while the invitation reached roughly 5500 CPs, many were not eligible because they do not conduct diagnostic assessments frequently enough to meet the inclusion criteria, which means that a conventional response rate cannot be calculated. The final sample of 93 clinicians who very regularly perform diagnostics is therefore appropriate for the study aims, although some degree of self-selection bias cannot be fully ruled out.

## Conclusion

In conclusion, the growing trend of self- and desired diagnoses is increasingly reflected in the daily practice of CPs, influencing both the course of assessments and the nature of patient–clinician interactions. This is particularly evident in the frequent requests for ADHD and ASD diagnoses, often connected to the wish for explanations of personal difficulties, a sense of belonging, or identification with narratives encountered in online communities. These motives reveal that, rather than being primarily treatment-oriented, diagnostic expectations often revolve around the identity-forming function of a diagnosis or the desire for a socially accepted framework to make sense of personal challenges. Additionally, the findings highlight that mismatches between anticipated and professional diagnoses can trigger intense responses from patients, which CPs must navigate while maintaining diagnostic integrity. Furthermore, patients with self- or desired diagnosis often require considerably more time and preparation, adding an extra layer of strain to the diagnostic process.

## Declaration of generative AI in scientific writing

During the preparation of this work the principal author used Open AI’s ChatGPT-5 in order to improve writing style and check grammar and spelling. After using this tool, all authors reviewed and edited the content as needed and take full responsibility for the content of the publication.

## Funding

This work was supported by Karl Landsteiner University of Health Sciences, Land Niederösterreich and Landesgesundheitsagentur Niederösterreich.

## Declaration of competing interest

Matthias Neumann, Verena Steiner-Hofbauer, Martin Aigner, Anna Höflich, Anita Holzinger, and Gloria Mittmann have no relevant financial or non-financial interests to disclose.

## Appendix Supplementary materials

Image, application 1

Image, application 2

Image, application 3
