# Supplementary material for: Increasing self- and desired psychiatric diagnoses among emerging adults: Mixed-methods insights from clinical psychologists
Source: Int J Clin Health Psychol. 2025 Dec 31;26(1):100661. doi: 10.1016/j.ijchp.2025.100661 (PMC12804154; doi:10.1016/j.ijchp.2025.100661)
Supplement: Supplementary file 1 [file mmc1.docx]

| **Supplementary Table 1** | | |
| --- | --- | --- |
| *Reported Characteristics of Patients with a Desired Diagnosis or Self-Diagnosis (N = 198)* | | |
| **Characteristic** | **Self-diagnosis** | **Desired diagnosis** |
| Higher Education | 18 | 22 |
| Female | 13 | 20 |
| High online activity | 8 | 17 |
| Job difficulties | 6 | 2 |
| Social life difficulties | 4 | 7 |
| Identity-related concerns | 4 | 5 |
| Performance pressure | 3 | 4 |
| Prior psychological history | 3 | 3 |
| High reported stress | 2 | 8 |
| Difficulties with life direction | 2 | 5 |
| Lower education | 2 | 4 |
| Difficulty assuming responsibility | 2 | 3 |
| Low self-esteem | 2 | 2 |
| High video game use | 2 | 1 |
| Substance abuse | 1 | 5 |
| Male | 1 | 4 |
| High socio-economic status | 1 | 3 |
| Psychological halfknowledge | 1 | 3 |
| Left-leaning political orientation | 1 | 1 |
| Low socio-economic status |  | 1 |
| Psychological Knowledge |  | 1 |
| Foreign nationality |  | 1 |
