# Supplementary material for: Increasing self- and desired psychiatric diagnoses among emerging adults: Mixed-methods insights from clinical psychologists
Source: Int J Clin Health Psychol. 2025 Dec 31;26(1):100661. doi: 10.1016/j.ijchp.2025.100661 (PMC12804154; doi:10.1016/j.ijchp.2025.100661)
Supplement: Supplementary file 2 [file mmc2.docx]

| **Supplementary Table 2** | | |
| --- | --- | --- |
| *Full Response Distributions for the Two Likert-Scale Items on Perceived Change in Self-Diagnoses and Desired Diagnoses* | | |
| **Response Option** | **Self-Diagnosis (n, %)** | **Desired Diagnoses (n, %)** |
| Much more often than before | 21 (22.6 %) | 26 (28.0 %) |
| More often than before | 47 (50.5 %) | 44 (47.3 %) |
| Same as before | 21 (22.6 %) | 20 (21.5 %) |
| Less than before | 0 (0 %) | 0 (0 %) |
| Much less than before | 0 (0 %) | 0 (0 %) |
| Never encountered | 4 (4.3 %) | 3 (3.2 %) |
