# Supplementary material for: Increasing self- and desired psychiatric diagnoses among emerging adults: Mixed-methods insights from clinical psychologists
Source: Int J Clin Health Psychol. 2025 Dec 31;26(1):100661. doi: 10.1016/j.ijchp.2025.100661 (PMC12804154; doi:10.1016/j.ijchp.2025.100661)
Supplement: Supplementary file 3 [file mmc3.docx]

| **Supplementary Table 3** | | | |
| --- | --- | --- | --- |
| *Robustness Analyses for Changes in Self-Diagnosed and Desired Diagnoses* | | | |
| **Analysis Condition** | **Test Type** | **Self-Diagnosis** | **Desired Diagnosis** |
| Main test | One sample *t* Test | *t*(92) = 13.08, *p* < .001,  *M* = 3.96, *SD* = 0.71 | *t*(92) = 13.65, *p* < .001,  *M* = 4.03, *SD* = 0.73 |
| Excluding “never encountered” | One-sample *t* test | *t*(88) = 13.656, *p* < .001, *M* = 4.00, *SD* = 0.69 | *t*(88) = 14.137, *p* < .001, *M* = 4.07, *SD* = 0.82 |
| Non-parametric check | Wilcoxon signed-rank | *W* = 2346, *p* < .001 | *W* = 2485, *p* < .001 |
| Distribution test | *χ*² goodness-of-fit test | χ²(4) = 83.075, *p* < 0.001 | χ²(4) = 75.781, *p* < 0.001 |
| *Note.* All tests support the same directional conclusion as the main analysis. | | | |
